# Trans-femoral versus trans-carotid access for transcatheter aortic valve replacement: an updated systematic review and meta-analysis

**DOI:** 10.2144/fsoa-2023-0101

**Published:** 2024-05-15

**Authors:** Naser Yamani, Syed Hasham Ali, Mahnoor Sadiq, Afeera B Ahmed, Kapil D Bhojwani, Vivek P Lohana, Saba Fatmah, Shazra Khalid, Hammad R Shamsi, Batool Zehra, Kaneez Fatima, Zulfiqar Q Baloch

**Affiliations:** 1 Division of Cardiology, University of Arizona Phoenix Medical Center, AZ 85721, USA; 2 Faculty of Medicine, Dow Medical College, Dow University of Health Sciences, Karachi, 74200, Pakistan; 3 Faculty of Medicine, Dow International Medical College, Dow University of Health Sciences, Karachi, 74200, Pakistan; 4 Faculty of Medicine, Karachi Medical & Dental College, Karachi, 74700, Pakistan; 5 Faculty of Medicine, Sindh Medical College, Jinnah Sindh Medical University, Karachi, 75510, Pakistan; 6 Division of Cardiology, Sparrow Hospital, Michigan State University, MI 48912, USA

**Keywords:** cardiology, cardiovascular, outcomes research, surgery

## Abstract

**Aim:** This meta-analysis aims to shed light on any primacy the *trans*-carotid (TC-TAVR) access may have over the *trans*-femoral access (TF-TAVR) for those undergoing transcatheter aortic valve replacement (TAVR). **Methods:** PubMed/MEDLINE and Cochrane Library were searched, from inception to March 2023 retrieving seven adjusted studies with a total of 6609 patients, of which 5048 underwent TF-TAVR while 1561 underwent TC-TAVR. **Results:** No divergence in risk of mortality, major bleeding or stroke/transient ischemic attack in TC-TAVR when compared with TF-TAVR was found. In TC-TAVR, the risk of vascular complications was low (OR: 0.51, 95% CI: 0.32–0.83, p = 0.003) as compared with TF-TAVR. **Conclusion:** As of this analysis, the viability of TC-TAVR as first alternative to TF-TAVR is plausible.

## Abstract (plain language summary)

This study represents the largest adjusted dataset to date, minimizing any confounding bias.

This study shows that there is no significant difference in 30-day mortality between the *trans*-carotid and transfemoral approach to TAVR.

This study shows that the *trans*-carotid approach is significantly protective against vascular complications.

We conclude that the *trans*-carotid approach is a safe first choice as an alternative when the *trans*-femoral approach is contraindicated.

Transcatheter aortic valve replacement (TAVR) is favored in older patients (over 80 years old) of any surgical risk category with life expectancy <10 years, and in older patients at high or prohibitive risk for mortality from SAVR with life expectancy of 1 year. SAVR is favored in younger patients (<65 years old) with life expectancy >20 years. TAVR has been approved by USFDA for patients at both the high risk and low risk end of the surgical risk spectrum, owing to excellent results in the PARTNER 3 and EVOLUT trials. These trials helped in expanding TAVR indication by showing that outcomes post-TAVR were superior to, or at least as good as, those post-SAVR in patients with severe aortic stenosis and low surgical risk [1,2]. According to AHA/ACC guidelines, TF-TAVR is recommended for symptomatic patients over the age of 80 with severe aortic stenosis, or younger patients with a life expectancy of fewer than 10 years and no anatomic contraindications so surgical AVR is supplanted by TAVR [3].

TF-TAVR technique is considered the gold standard in TAVR patients as recommended by the European Society of Cardiology (ESC) and the European Association of Cardiothoracic Surgery (EACTS) [4]. It accounts for an estimated 80–85% of all TAVR cases [5,6]. However, TF-TAVR is contraindicated in peripheral vascular disease – particularly vessels which have a diameter <5.5 mm, are tortuous, or have moderate to heavy vessel calcification [7]. Alternative access sites have been used in certain circumstances, including *trans*-axillary (TAx), transapical (TA), transaortic (TAo) and *trans*-carotid (TC) [8]. When an alternative TAVR procedure is likely to be required, TC access is concurrently being considered as the primary substitute due to the ease with which the aortic annulus can be reached through this approach [9,10]. However, current evidence comparing the safety and efficacy of TC-TAVR with TF-TAVR is limited, with mostly small-sized studies with varying findings. To resolve these inconsistencies and provide a holistic comparison of the two approaches, we conducted a systematic review and meta-analysis of all adjusted studies to date.

## Methods & materials

This meta-analysis was conducted following the Preferred Reporting Items for Systematic Review and Meta-Analysis (PRISMA) guidelines [11]. As this is a compilation of publicly accessible results, no institutional review board permission (IRB) or patient informed consent was required.

### Eligibility criteria

Abstracts, case studies, case reports, review articles, meta-analyses, non-human studies, and publications in languages other than English were not considered. The population of interest was patients with Aortic Valve Stenosis. The intervention was the TC-TAVR group; the control was the TF-TAVR group, and the outcome was assessing the safety of the TC-TAVR route. Studies were incorporated if they met the accompanying criteria: 1) original articles, 2) TC-TAVR versus TF-TAVR, 3) published information on population characteristics, periprocedural outcomes and 30-day clinical outcomes, and 4) had adjusted outcome values reported in ORs. The authors consider it important to mention that Villecourt *et al.* [12] was not included in this meta-analysis as, although it did have a propensity matched population, it pooled results of both the TC and *trans*-subclavian (TSc) approaches.

### Literature search process

PubMed/Medline and Cochrane Library were searched from the inception of databases until 24 March 2023. Grey literature was also searched using Google Scholar and all the significant studies were included using the appropriate keywords and MeSH terms, (Transcatheter Aortic Valve Replacement OR Percutaneous aortic OR Transcatheter aortic valve implantation OR TAVI OR TAVR) AND (*trans*-carotid OR transcervical OR *trans*-carotid OR carotid OR nonfemoral OR non-femoral) AND (Transfemoral OR *trans*-femoral OR femoral) (Supplementary Table 1). Filters were not applied during the literature search nor were any language or time limitations. To expand our literature search, all the keywords were screened for the titles and the abstracts. All the selected articles were then run in EndNote, and any duplicates were identified and removed. The selected articles were screened on three levels: title, abstract and full text by two independent reviewers (A.B.A and S.H.A). This process yielded 14 articles, of which seven were adjusted and were finally analyzed to reduce confounding bias.

### Outcomes of interest, data extraction & risk of bias assessment

Pooled dichotomous outcomes included 30-day all-cause mortality, 30-day vascular and major vascular complications, major bleeding, permanent pacemaker implantation procedure (PPM), cardiac tamponade and 30-day stroke/TIA. Furthermore, the one continuous outcome analyzed was hospital stay. For each outcome, a subgroup analysis was performed with propensity-score matched studies using p-values. Outcomes were defined as reported in the studies; whenever possible, the Valve Academic Research Consortium-2 (VARC-2) definitions were used [13].

A predesigned Excel spreadsheet was used to extract study data and baseline characteristics. Quality assessment of included studies was conducted using the Newcastle–Ottawa scale [14], independently by two reviewers (M. S. and K.D.B), and inconsistencies were determined via discussion.

### Statistical analysis

We used Open Meta-Analyst and Review Manager Version 5.0 (The Cochrane Collaboration, The Nordic Cochrane Centre, Copenhagen, Denmark) for all statistical analyses. Odds Ratios (OR) and corresponding 95% CI of all dichotomous clinical outcomes were extracted and analyzed. For the one continuous outcome included in the study, medians and interquartile ranges (IQR), standard mean difference (SMD), weighted mean difference (WMD) and frequencies with percentages were used and corresponding 95% CI was calculated as well. All data were extracted either by reviewing tables or figures in studies directly, or imputed to ORs using the Review Manager software when data were given in frequencies and percentages.

Potential confounders were adjusted by using adjusted data, PS weighted data and multivariate regression analysis. A random-effects model was utilized to account for the anticipated heterogeneity, procedural discrepancies, and certain outcome definitions. Weightage of all non-dichotomous and dichotomous outcomes was assigned using inverse variance. The Higgins (I^2^) statistic was used to evaluate heterogeneity and a value of 25%–50% was considered mild, 50%–75% as moderate, and >75% as severe heterogeneity. Publication bias was assessed through visual inspection of funnel plots. P-value ≤0.05 was regarded as significant for all analyses (Supplementary Figures 1–7).

### Data availability

Data regarding this meta-analysis which is not already divulged herein are available from the corresponding author on reasonable request.

## Results

### Literature search results synthesis

**Figure 1.**
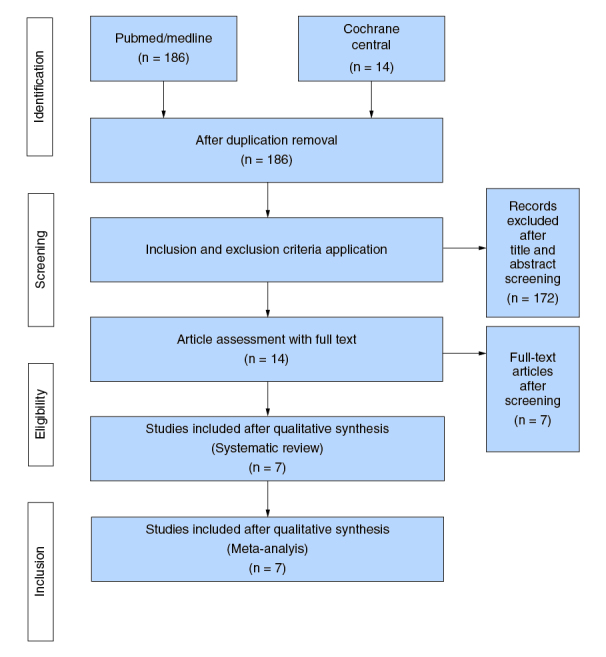
PRISMA flowchart.

Our literature search yielded 186 articles, of which 14 studies [^5-7^,^12^,^15-24^] remained after exclusion. Seven of the 14 were adjusted and thus finally analyzed [6,^16-21^]. The PRISMA flowchart (Figure 1) summarizes the results of our literature search.

### Study characteristics & quality assessment

According to the Newcastle–Ottawa Scale [14], all studies had a high methodological quality (Supplementary Table 2).

### Patients' baseline characteristics

A total of seven studies included 6609 patients which consisted of 5048 patients undergoing TF-TAVR and 1561 patients undergoing TC-TAVR. Compared with those undergoing TF-TAVR, patients undergoing TC-TAVR were younger (Mean Difference -0.89 95% CI: [-1.41, -0.38]), and had a significantly greater prevalence of peripheral vascular disease (56% vs 29%), prior cardiac surgery (26% vs 20%) and diabetes (35% vs 33%). This can be explained by shared risk factors as highlighted by studies such as PARTNER-B and CoreValve [25].

There was no significant difference regarding permanent pacemaker implantation (PPM), preoperative stroke/TIA, atrial fibrillation, arterial valvular surface (AVS), and pre-operative creatinine levels.

Surgical risk in both approaches was statistically insignificant when the logistic Euroscore, Euroscore II and STS-PROM score was used as a metric. A detailed analysis of dichotomous and continuous baseline characteristics is outlined in the tables below (Tables 1 & 2). Surgical risk scores of both cohorts have been detailed in Supplementary Table 3.

**Table 1.** Patient characteristics (dichotomous).

Patient characteristics (dichotomous)								
Variables	Studies (n)	Events in TC group	Total number of patients (TC)	Events in TF group	Total number of patients (TF)	Odds ratio, M–H random, 95% CI	p-value	Heterogeneity (I^2^) (%)
Males	7	894	1561	2758	5048	1.08 [0.86, 1.36]	0.48	61
Hypertension	6	980	1499	3723	4986	1.17 [0.85, 1.61]	0.34	56
COPD/chronic lung disease/respiratory faliure	6	418	1461	1174	4648	1.46 [0.96, 2.21]	0.08	85
Diabetes	7	542	1561	1677	5048	1.21 [1.04, 1.40]	0.01	16
Cerebrovascular disease	3	49	358	228	2160	1.43 [0.99, 2.08]	0.06	13
Peripheral vascular disease	7	868	1561	1464	5048	4.75 [1.55, 14.56]	0.006	98
NYHA functional class III/IV	5	303	520	1501	2622	1.02 [0.83, 1.25]	0.88	2
Stroke/TIA	5	158	1287	566	4145	0.87 [0.64, 1.18]	0.37	39
Prior cardiac surgery	2	282	1057	594	2932	1.46 [1.02, 2.09]	0.04	66
Permanent pacemaker implantation	3	151	1073	279	2075	1.01 [0.78, 1.29]	0.96	5
Atrial fibrillation	5	209	550	1233	3035	0.92 [0.76, 1.11]	0.39	0

**Table 2.** Patient characteristics (continuous).

Patient characteristics (continuous)						
Variables	Studies (n)	Total number of patients (TC)	Total number of patients (TF)	Mean difference, IV, random, 95% CI	p-value	Heterogeneity (I^2^) (%)
Age (years)	6	1434	4649	-0.89 [-1.41, -0.38]	0.0006	4
BMI	5	523	3036	-0.26 [-1.00, 0.48]	0.48	12
Creatinine	3	331	2161	-0.00 [-0.17, 0.17]	1.00	0
LVEF	6	1434	4649	-0.32 [-1.54, 0.89]	0.60	42
Aortic valvular mean gradient	4	1287	4145	-1.08 [-3.26, 1.10]	0.33	74
Aortic valve surface	5	1372	4587	-0.01 [-0.02, 0.00]	0.18	0

### Results of meta-analysis

Thirty-day complications of TC-TAVR and their respective incidences in adjusted studies are analyzed and presented below. An exact count of the number of incidences could not be given in most cases as most studies solely reported adjusted ORs and did not divulge the exact patient count in each cohort.

#### 30-day all-cause mortality

**Figure 2.**
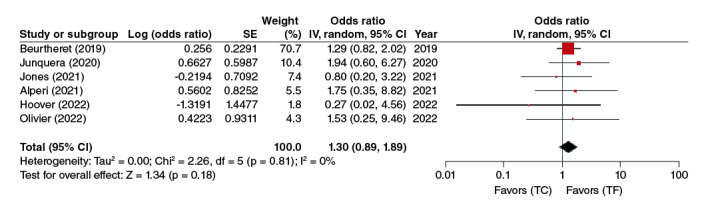
30 day all-cause mortality forest plot.

In a pooled analysis of 6 adjusted studies, this association was shown to be non-significant (OR: 1.30, 95% CI: 0.89–1.89, p = 0.18), where the risk of mortality was similar in TC-TAVR when compared with TF-TAVR (Figure 2).

#### Vascular complications

**Figure 3.**
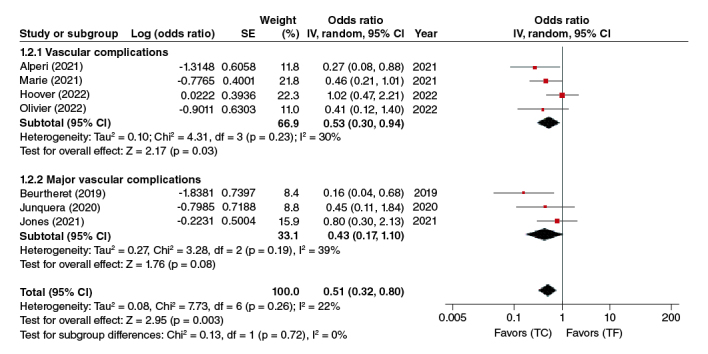
Vascular complications forest plot.

In the pooled analysis of the four adjusted studies; analysis of vascular complications and three adjusted studies subgroup analysis of major vascular complications, this association was statistically significant (OR: 0.51, 95% CI: 0.32–0.80, p = 0.003). TC-TAVR was thus associated with significantly fewer vascular complications (Figure 3).

#### Major bleeding

**Figure 4.**
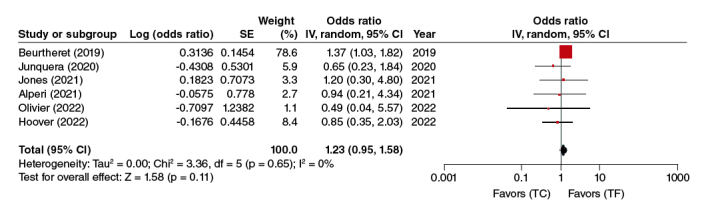
Major bleeding forest plot.

In the pooled analysis of the six adjusted studies, the TC-TAVR approach was not significantly associated with a higher risk of major bleeding (OR: 1.23, 95% CI: 0.95–1.58, p = 0.11) (Figure 4).

#### Permanent Pacemaker Implantation

**Figure 5.**
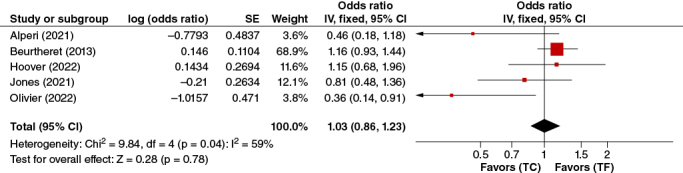
Permanent pacemaker forest plot.

In the pooled analysis of five adjusted studies, no significant association between permanent pacemaker implantation and TC-TAVR or TF-TAVR was found. (OR: 1.03, 95% CI: 0.86–1.23, p = 0.78) (Figure 5).

#### Cardiac tamponade

**Figure 6.**
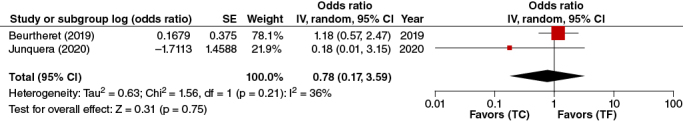
Cardiac tamponade forest plot.

In the analysis of the pooled two adjusted studies, no statistical difference was observed between TC-TAVR and TF-TAVR (OR: 0.78, 95% CI: 0.17–3.59, p = 0.75) (Figure 6).

#### 30-day stroke/TIA

**Figure 7.**
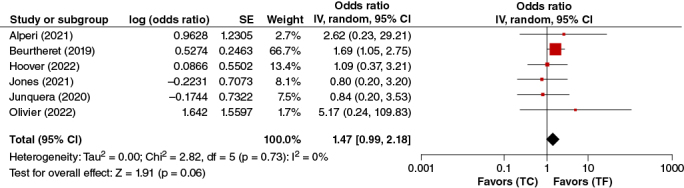
30 day stroke/TIA forest plot.

In the pooled analysis of the five adjusted studies reporting data on 30-Day Stroke/TIA, TC-TAVR was not significantly associated with a higher risk of 30-Day Stroke. (OR: 1.47, 95% CI: 0.99–2.18, p = 0.06) (Figure 7).

#### Hospital stay

**Figure 8.**
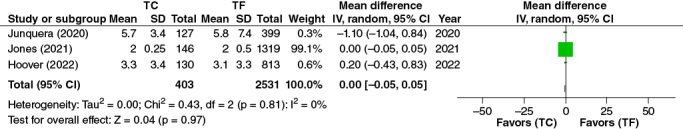
Hospital stay forest plot.

In the pooled analysis of the three adjusted studies wherein 403 patients were accounted for in TC-TAVR, and 2531 patients were accounted for in TF-TAVR, no statistical difference was observed in duration of hospital stay (Mean Difference, 0.00, 95% CI: -0.05–0.05, p = 0.97) (Figure 8).

## Discussion

The choice of alternative TAVR access in patients who are not eligible for TF-TAVR is debatable. The *trans*-apical (TA-TAVR) procedure was the first alternate approach for TAVR, but its use has gradually reduced among TAVR operators due to mounting evidence of the strategy's increased risk of problems, such as higher peri and postoperative mortality [26,27]. Recently, TSc and TC alternate access approaches have developed and substituted TA-TAVR access in numerous centers [^28-31^]. The TC approach has also shown promise when compared with TA and TAo approaches, with better 2-year survival outcomes and a shorter hospital length-of-stay [32].

Our meta-analysis and systematic review represent the largest adjusted dataset to date comparing TC-TAVR to TF-TAVR. Studies have suggested that these two pathways yield slightly different outcomes. Our analysis demonstrates the following findings: 1) TC-TAVR and TF-TAVR accesses didn't yield any significant difference regarding 30-day all-cause mortality. 2) TC-TAVR patients presented a lower risk of vascular complications, and 3) TC-TAVR and TF-TAVR didn't yield any significant difference between approaches when considering other post-operative outcomes (PPM, Cardiac Tamponade, Hospital Stay, Major Bleeding, and 30-Day Stroke/TIA).

We found no statistically significant difference between TC-TAVR and TF-TAVR in 30-day all-cause mortality. According to data reported in prior meta-analyses published by Lu *et al.* and McGrath *et al.*, there was no significant correlation between the TC-TAVR and the TF-TAVR group's mortality statistics [8,33]. The findings did confirm that TC-TAVR may serve as a viable alternative for TF-TAVR in the proper clinical setting and patient selection. Admittedly, the most recent meta-analysis by Abraham *et al.* did find that TC-TAVR patients had significant higher 30-day all-cause mortality which it explained with a higher co-morbidity burden in the TC-TAVR cohort [34]. However, post-adjustment for confounding factors, mortality data is statistically insignificant in our study, and is back in concordance with previous adjusted datasets.

Our study makes the point that the TC-TAVR approach is associated with a lower risk of major vascular complications. In the PARTNER trial aimed at finding the incidence of major vascular complications, the study concluded them to be relatively prevalent after TF-TAVR and associated with higher 30-day all-cause mortality [35]. Previous two meta-analyses published also report similar findings of TC-TAVR being associated with a lower risk of major vascular complications [8,33]. Furthermore, a meta-analysis by Usman *et al.* evaluating the safety of TC-TAVR also shows it to be associated with lower major vascular complications [36]. These results make for a strong case for the use of the TC-TAVR approach in patients at risk of developing such complications. Previous meta-analyses have similarly revealed a lower incidence of vascular complications with TC-TAVR [34,36]. One explanation could be that the common carotid artery is approached, cannulated and reconstructed surgically in TC-TAVR, but most TF-TAVR cases are treated percutaneously, which does not allow direct vascular control [33,37].

Stroke complications during the TC-TAVR procedure, according to Mylotte *et al.* [38] may be caused by:

▪ Embolization of carotid artery plaque due to arterial puncture and instrumentation;
▪ Access site trauma providing a nidus for thrombosis with subsequent embolization;
▪ Inadequate collateral perfusion through the circle of Willis;
▪ Embolization of debris during balloon valvuloplasty or transcatheter heart valve implantation.

Folliguet *et al.* looked at studies that reported strokes, but they could not find a correlation with the site of the access [39]. Our study also found no statistically significant difference between the two approaches in terms of stroke/TIA risk. At the same time however, certain steps can be taken to further reduce the number of cerebrovascular events by following the key approach that is:

▪ A proper patient eligibility evaluation based on Cardiac imaging by multi-slice computed tomography (MSCT) findings;
▪ Selection of a larger diameter artery;
▪ Lower tortuosity;
▪ Being less calcified;
▪ Having a more favorable spatial relationship between the virtual common carotid artery centerline and the plane of the aortic annulus;
▪ All patients undergoing before the intervention, TC-TAVR were carefully screened for common carotid artery atherosclerotic plaques;
▪ The functional integrity of the circle of Willis was systematically assessed intraoperative using the common carotid artery clamping test;
▪ At the end of interventions, the access artery was reconstructed using interrupted sutures, allowing for back-bleeding purging of intravascular debris [22].

A study by Tsai *et al.* proposes a double sheath connection technique as a way to possibly reduce the risk of stroke [40]. As suggested by Kahlert *et al.* [41], the use of a cerebral embolic protection (CEP) device during TC-TAVR may further reduce the risk of stroke or transient ischemic attack (TIA).

It is pertinent to note that the previous meta-analysis which reported adjusted outcome values, Lu *et al.* [33], highlighted a significant risk of neurovascular complications in the TC-TAVR approach [33]. A divergence in results can be explained by the fact we had more studies which formed a larger, more representative dataset.

This meta-analysis reports TC-TAVR to be associated statistically non significantly with major bleeding as a 30-day outcome when compared with TF-TAVR. Pharmacological options can also be exercised to reduce bleeding, such as administration of recombinant Factor VIIIa, however it should be noted that further RCTs are required to establish if such interventions are safe to use as either first line therapies for bleeding or bleeding prophylaxis [42]. However, owing to the nature of the incisions it is likely that bleeding would be more mechanical in origin. Additionally, we do appreciate that the possibility of a thrombus forming close to the brain or a serious bleed occurring because of the procedure may be cause for apprehension, but it can be allayed by the fact that our analysis did not find bleeding incidences to be statistically significant. Thrombus formation was not seen as a considerable risk in the analyzed studies either.

Furthermore, it is noteworthy that PPM rates between the two access routes are statistically insignificant. The PARTNER trial though, does conclude that generally patients undergoing TAVR have higher implantation rates than those undergoing SAVR [43].

The carotid artery has been used frequently as another approach for TAVR. The study's purpose was to compare the convenience of *trans*-carotid (TC) versus *trans*-femoral (TF) TAVR [24]. The gold standard approach is TF-TAVR but in conditions where this route is contraindicated, TC-TAVR has been shown to be a viable alternative. The possible explanations embody the continued advances in TAVR technology with the event of newer-generation valves with higher deliverability, lower profile, a rise in operator expertise, and additionally evolving modalities of screening of patients appropriate to the current approach. Furthermore, whether the distinction concerning the categories of THV (*trans*-catheter heart valves) used between TC-TAVR and TF-TAVR could have wedged the outcomes, is unclear [33]. Additional literature and specific statistics are required to identify the patients at higher risk for major vascular complications and sudden vascular repairs who would gain benefit from the alternative access i.e. TC-TAVR [21]. Limited data are available comparing post-procedural outcomes among TC-TAVR and TF-TAVR which includes procedural mortality and percentage of procedural success regardless of the high-risk profile of TC patients. Generally, our results signify the importance of careful peri-procedural patient selection which in turn would help in specifying the TC or TF route as suited. Studies to outline the choice criteria of TC-TAVR are still required [33].

Our study showed TC-TAVR is associated with a lower risk of vascular and major vascular complications as opposed to TF-TAVR. On the other hand, our study showed TC-TAVR akin to TF-TAVR regarding 30-day all-cause mortality, 30-day stroke/TIA, major bleeding, permanent pacemaker implantation, cardiac tamponade and hospital stay.

## Conclusion

Overall, this meta-analysis concluded that the TC-TAVR can be a safe alternative to TF-TAVR, owing to its decreased risk of vascular complications and a statistical insignificance in other outcomes. However further research must be conducted to establish its safety and efficacy in its interim state.

## Limitations

Our systematic review and meta-analysis have limitations that must be taken into consideration. Firstly, there were no randomized controlled trials (RCTs), so findings were derived from observational studies. Since TF-TAVR is a preferred approach for TAVR, the chances of RCTs being performed in theory are slim. Secondly, all of the studies were retrospective in nature that are subject to recall bias. A lack of standardization in both baseline and outcome characteristics, along with unreported data in different outcomes by the included studies has led to the presence of some confounding bias. Furthermore, our study had possible publication bias, as suggested by the funnel plot (Supplementary Figures 1–7). The authors consider it important to mention the fact that the studies included in this meta-analysis did not differentiate between current patient gender identity and sex assigned at birth.
